# Complex Microphthalmia in Three Central Asian Shepherd Dogs

**DOI:** 10.1002/vms3.70494

**Published:** 2025-08-19

**Authors:** Reza Azargoun, Seyed Mohammad Hashemi‐Asl, Shokoufa Deldar

**Affiliations:** ^1^ Department of Internal Medicine and Clinical Pathology Faculty of Veterinary Medicine Urmia University Urmia Iran; ^2^ Department of Surgery and Diagnostic Imaging Faculty of Veterinary Medicine Urmia University Urmia Iran

**Keywords:** canine, cataract, ectopia lentis, microphthalmos, ophthalmology

## Abstract

Congenital eye malformations are uncommon and in some dog breeds, there is no evidence of their occurrence. This report aimed to describe the clinical and ultrasonographic findings of complex microphthalmia in Central Asian Shepherd dogs. Three 2‐month‐old female Central Asian Shepherd puppies from two litters were referred to our teaching hospital with the owner's complaint of eye abnormalities since birth. The puppies were alert on clinical examination, the vital signs were normal, and no other structural abnormalities were observed. In the ophthalmological assessments of all three dogs, the globe appeared bilaterally small and sunken in the orbit. In the ultrasound of the puppies’ eyes, the lens thickness and axial length of the globe were greater and less than the values measured in the eyes of a healthy puppy of the same age and breed, respectively. Moreover, in all three puppies, the lenses were located in the vitreous chamber and displaced perpendicular to their natural axis. On the basis of this, bilateral complex microphthalmia and congenital ectopia lentis, two ocular malformations of unclear etiology, were diagnosed. Due to the possibility of an association between such malformations and a high grade of inbreeding in kennels, as well as environmental and genetic factors, these conditions can be limited by breeding efforts and pre‐breeding screening plans.

## Graphical abstract

Here is a description of the main ultrasound findings in three Central Asian Shepherd puppies with complex microphthalmia, a rare congenital ocular malformation, compared to a healthy puppy of the same breed, age and sex. Accordingly, in dogs with complex microphthalmia, the mean ± standard deviation of axial length (AL) of the globe was shorter, and the vitreous chamber depth (VCD) and lens thickness (LT) were greater than those of the healthy dog. Surprisingly, all patients had ectopia lentis (EL).

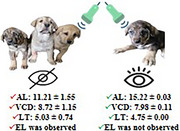

## 1 Introduction

Microphthalmia is one of the most drastic developmental eye anomalies that may have devastating effects on vision (Plaisancié et al. 2019; Murgiano et al. 2024). This anomaly is characterized by unilateral or bilateral small‐globe and is divided into simple or complex types (Shaw et al. 2019; Plaisancié et al. 2019). Simple microphthalmia implies anatomically intact eye(s) with reduced size. Oppositely, complex microphthalmia represents the reduced size of eye(s) along with disorders of the eye's anterior section (e.g., cataract) and/or posterior section (e.g., retinal dysplasia) (Plaisancié et al. 2019). The etiology of microphthalmia is not fully understood; however, it may be associated with environmental and genetic factors. Environmental factors, as a minor cause, can include infectious (e.g., toxoplasmosis) and noninfectious agents (e.g., NSAIDs use). Genetic factors are recognized as probable major causes and hundreds of genes may be involved in the eye development of humans or animals (Plaisancié et al. 2019).

Microphthalmia has been recognized in several dog breeds, including, but not limited to, Australian Shepherd, Akita, Bedlington Terrier, Beagle, Collie, Cavalier King Charles Spaniel, Doberman Pinscher, Dachshund, Miniature Schnauzer, Great Dane, Poodle, Old English Sheepdog, Soft‐Coated Wheaten Terrier, Saint Bernard and more commonly in mixed breeds (Murgiano et al. 2024). It seems that no report of complex microphthalmia in the Central Asian Shepherd dog breed has been published so far, and we intend to describe the clinical and ultrasonographic findings of this congenital anomaly in three Central Asian Shepherd puppies.

## 2 Case Report

**FIGURE 1:**
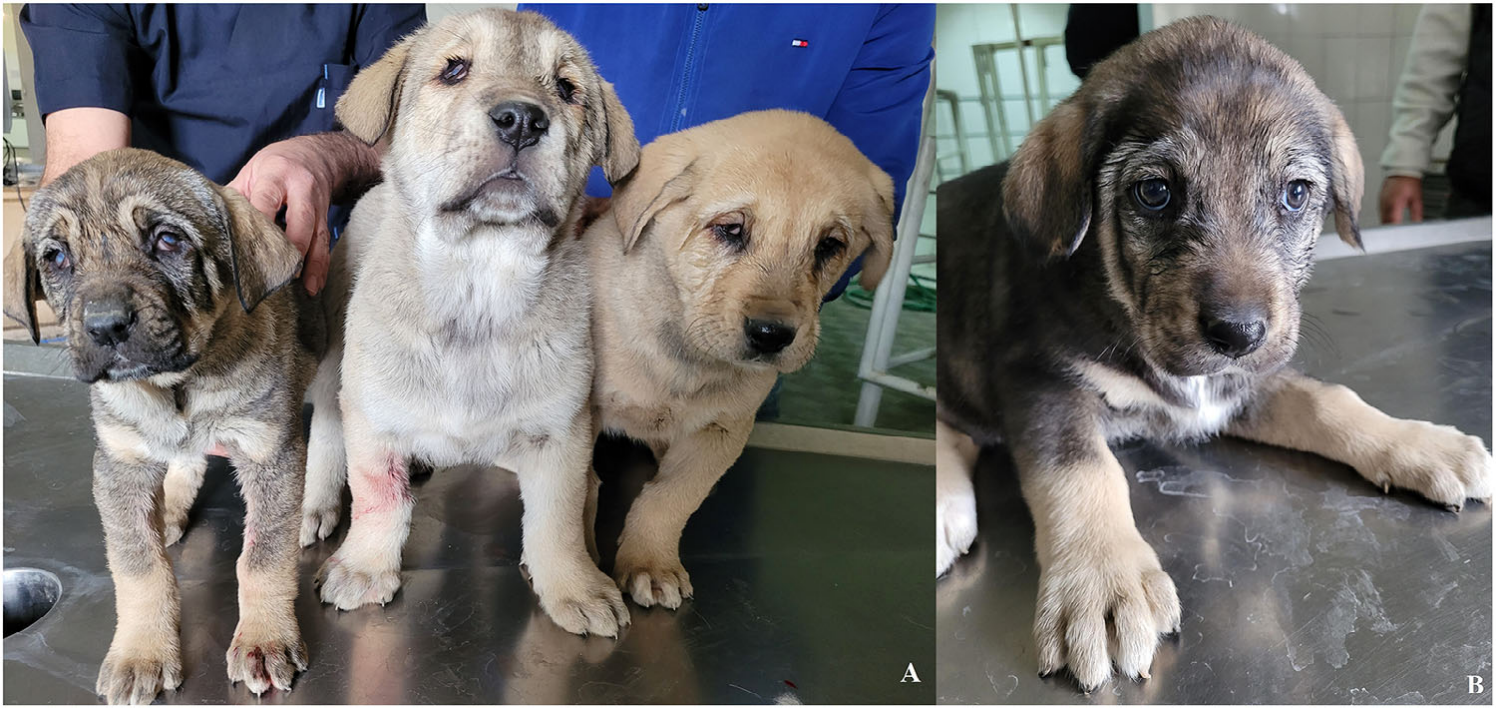
(A) The Central Asian Shepherd dogs with complex microphthalmia; From right to left: puppy number one, puppy number two and puppy number three. (B) A healthy Central Asian Shepherd dog with normal eyes; Puppy number four.

The owner consented to using his puppies’ data in scientific papers. Three 2‐month‐old female puppies of the Central Asian Shepherd dog breed were referred to Urmia University Veterinary Teaching Hospital with the owner's complaint of ocular malformations since birth (Figure 1A). Two of the puppies (numbers one and two) were from the same parents, and the third was from different parents, also these parents and other littermates were healthy according to the owner's claim. The patients had received a full course of vaccinations and antiparasitic treatment. On physical examination, the puppies were alert, the vital signs were normal, and no other structural abnormalities were observed. Head palpation revealed that both globes of the puppies were subjectively small. Ophthalmological examination revealed that the eyelashes had developed normally; however, the sunken eyelids and reduction of the palpebral fissures precluded a full inspection of the ocular structures. In all puppies, the palpebral reflex was intact, the menace response was absent, and the pupillary light reflex was not interpretable due to corneal opacity. The mean values of Schirmer's tear test and intraocular pressure were 15.2 mm/min and 12.7 mm Hg, respectively, as well as the fluorescein staining of the cornea was negative in both eyes of the puppies. Moreover, the haematological parameters were unremarkable.

**FIGURE 2:**
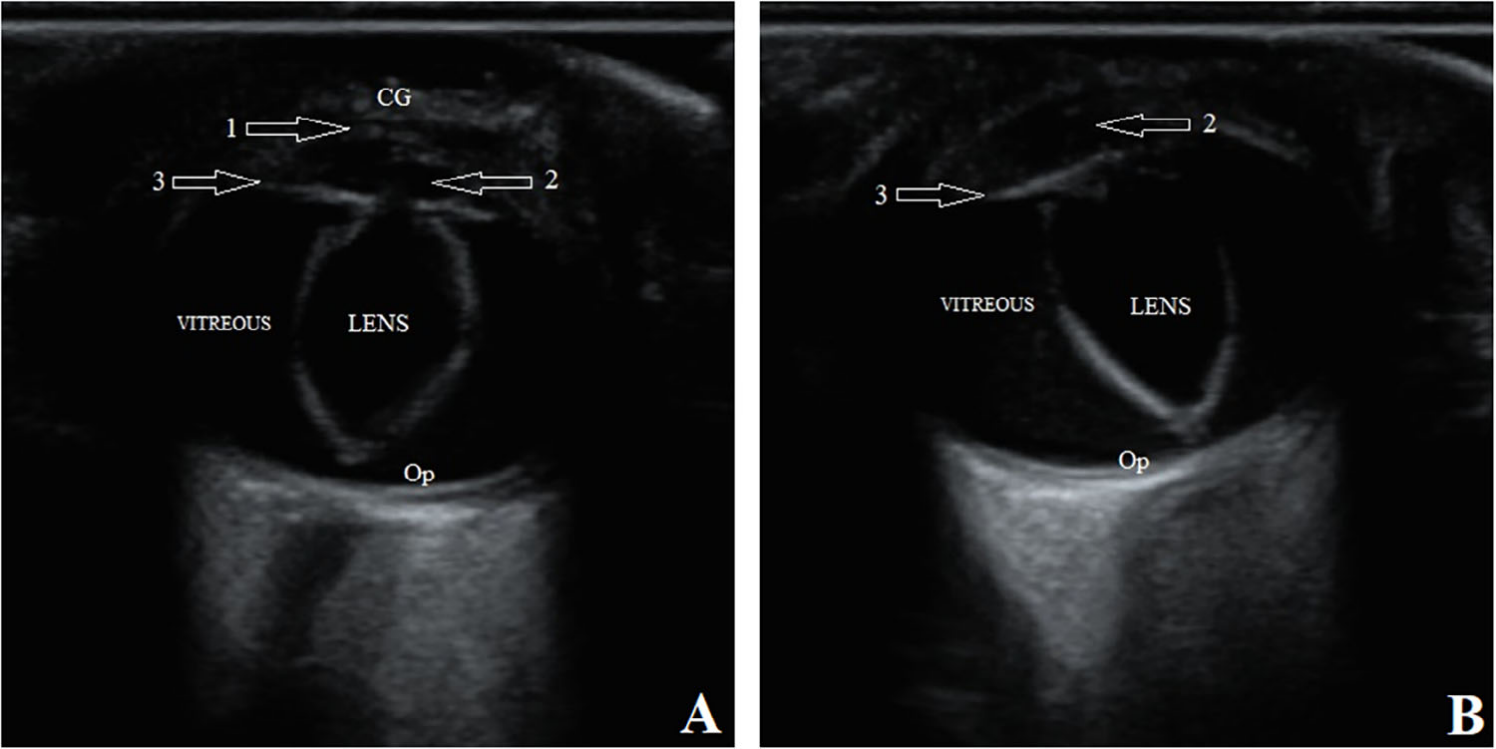
B‐mode ultrasound images of complex microphthalmia in the right (A) and left (B) eyes of puppy number one. Arrows: 1, cornea; 2, anterior chamber; 3, ciliary body. CG, coupling gel; Op, optic disc.

**FIGURE 3:**
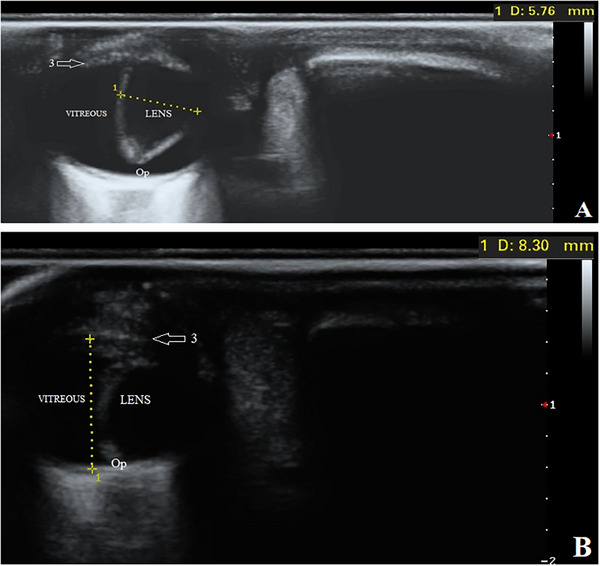
B‐mode ultrasound images of complex microphthalmia in the right (A) and left (B) eyes of puppy number two. Arrow: 3, ciliary body. Op, optic disc.

**FIGURE 4:**
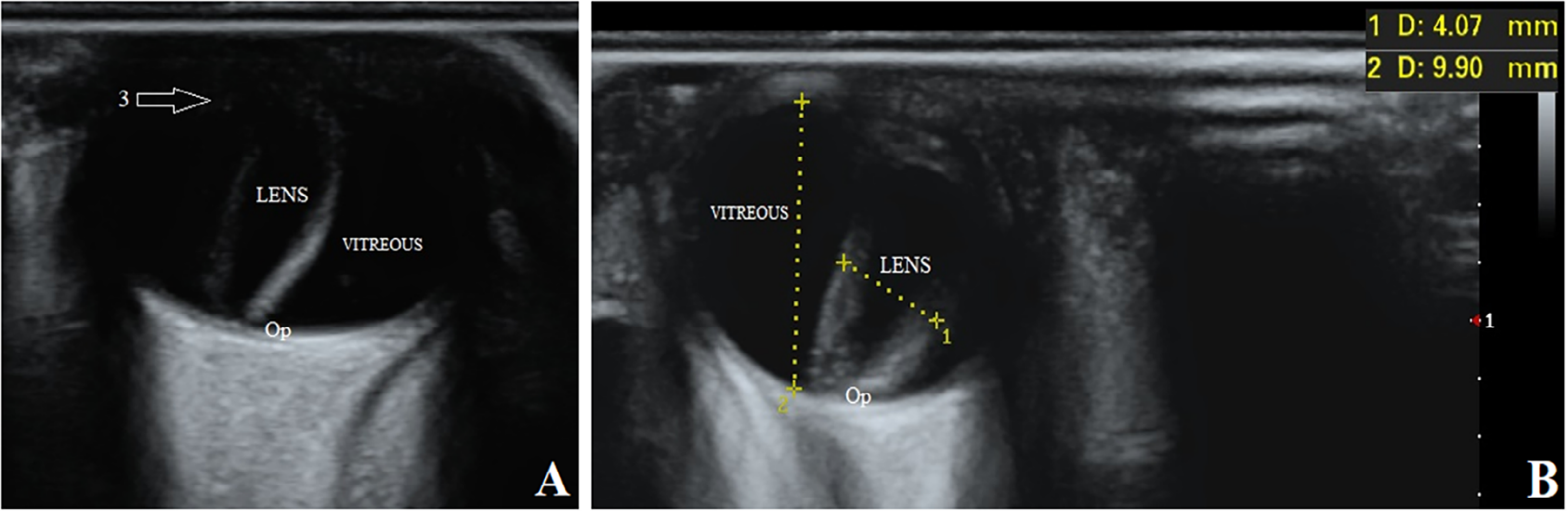
B‐mode ultrasound images of complex microphthalmia in the right (A) and left (B) eyes of puppy number three. Arrow: 3, ciliary body. Op, optic disc.

**FIGURE 5:**
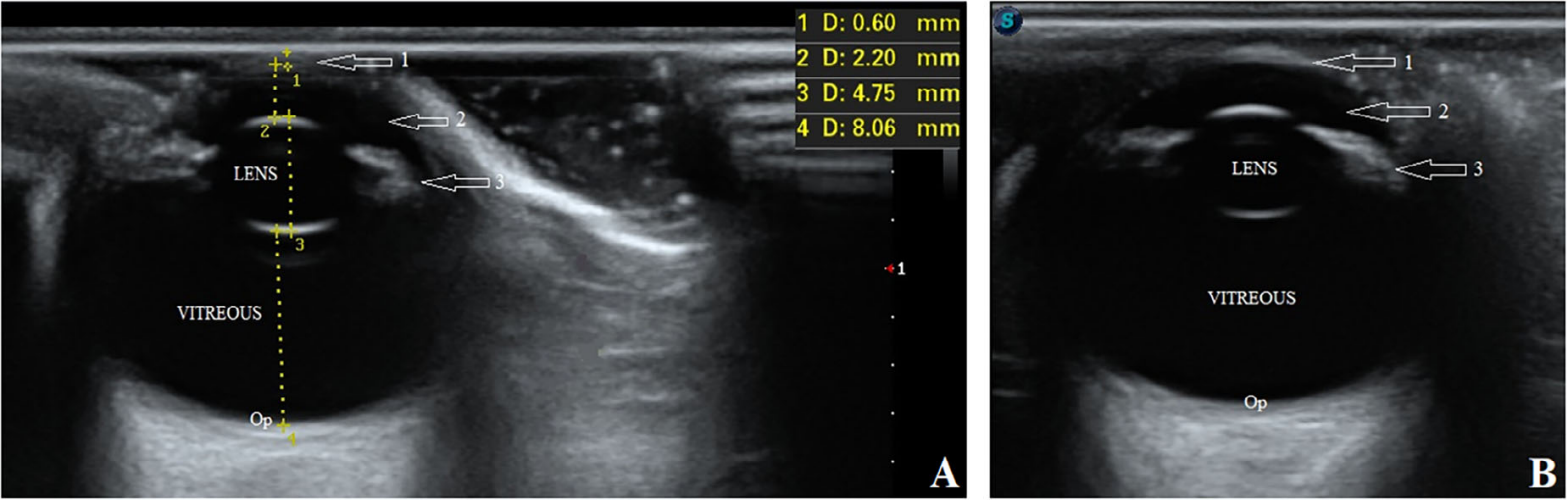
B‐mode ultrasound images of the right (A) and left (B) eyes of puppy number four. Arrows: 1, cornea; 2, anterior chamber; 3, ciliary body. Op, optic disc.

Then, the pups underwent an ultrasonographic examination of both eyes. The transpalpebral technique was done using an ultrasound machine (SonoScape P40, Shanghai, China) with an 8–13 MHz linear transducer and coupling gel. Although the puppies were restrained in a sitting position, the eyes were anaesthetized with 0.5% Tetracaine eye drops (Anestocaine, Sinadarou, Tehran, Iran) and scanned in the sagittal and dorsal planes. The anterior structures of the eyes, such as the cornea, anterior chamber and ciliary body, were almost identifiable in pups number one and two. However, in pup number three, the mentioned structures were not well visible. The characteristics and echogenicity of the vitreous, retina and optic disc were almost normal in all three puppies. Surprisingly, displacement of the lens with a rotation of about 90 degrees was observed in all patients. The lenses were in the vitreous chamber (i.e., Ectopia lentis) so that the equatorial margin was close or in contact with the optic disc (Figures 2, 3, 4). To more accurately evaluate the observed ocular malformations, a healthy dog of the same breed, age and sex (puppy number four) (Figure 1B) was also subjected to ocular ultrasonography (Figure 5). Compared to the healthy dog, the patients’ mean values of globe axial length and lens thickness showed a decrease (15.22 ± 0.03 vs. 11.21 ± 1.55 mm) and an increase (4.75 vs. 5.03 ± 0.74 mm), respectively. The values of ocular structures measured by ultrasound are presented in Table 1. According to clinical and ultrasonographic findings, bilateral microphthalmia and ectopia lentis were confirmed.

**TABLE 1:** The measurable ultrasound values of ocular components in the Central Asian Shepherd dogs with complex microphthalmia (puppies 1–3) compared to a healthy dog (puppy 4).

	Eye components (mm)							
Puppy number	Anterior chamber depth		Lens thickness		Vitreous chamber depth		Axial length	
	OD	OS	OD	OS	OD	OS	OD	OS
1	2.02	2.31	5.46	5.75	9.05	9.44	11.07	12.66
2	^a^	2.15	5.76	4.23	8.30	6.95	^a^	9.10
3	^a^	^a^	4.96	4.07	^a^	9.90	12.02	^a^
4	2.20	1.95	4.75	4.75	8.06	7.90	15.20	15.25

*Note*: OD, right eye; OS, left eye.

^a^ It was not measurable.

## 3 Discussion

The globe formation is due to coordinated induction and differentiation processes in the embryonic period (Plaisancié et al. 2019). The development process of dogs’ eyes is similar to that of humans, and the main difference is the time of some developmental events (Shaw et al. 2019). Disturbance in any of these events can potentially cause eye developmental and structural anomalies such as microphthalmia. It has been suggested that microphthalmos may be caused by a lack of induction at the level of the primary neural tube, the inability of the optic pit to enlarge and form the optic vesicle, or secondary regression of the ocular structures during the developmental period (Plaisancié et al. 2019). Of note, although 29 different mutations are known to be associated with hereditary ocular lesions in dogs, none have been recognized for microphthalmia (Shaw et al. 2019).

Microphthalmos is a rare condition, and little research has been conducted on this topic (Sandhu et al. 2020). In humans, this condition affects 3%–11% of blind neonates, with an incidence of almost 19 per 100,000 live births (Berkowski et al. 2018). In an epidemiological study of a population of 32,974 dogs over 7.5 years, the prevalence of microphthalmia among all diagnosed congenital ocular abnormalities with an overall incidence of 0.3% was reported to be 35%. Although our patients were 2‐month old and female, in Saraiva and Delgado's study (2020), the median age of dogs with microphthalmia was 7.5 months, and similar to humans, sex predisposition was not statistically significant; however, most of the patients were male. In the current study, B‐mode ultrasonography, as a proven method for biometric measurements, was used for a detailed evaluation of globe axial length and eye internal structures (Plaisancié et al. 2019). Though the reference values for the ocular components of Central Asian Shepherd dogs have not been established yet, in Tuntivanich et al.’s study (2007), the mean of globe axial lengths in 2‐month‐old normal mesocephalic cross‐bred dogs was reported to be about 15.55 mm. This reported value and what was recorded from the ultrasonographic examination of the eyes of puppy number four showed higher values than the mean of globe axial lengths of our patients (11.21 mm), which can indicate the small size of their globes (i.e., microphthalmia).

In human ophthalmology, a congenital eye malformation does not often occur alone, particularly when developmental errors arise early in embryonic growth. Nevertheless, it has been suggested that congenital eye malformation often occurs alone in dogs (Saraiva and Delgado 2020). Surprisingly, in addition to microphthalmos, the Central Asian Shepherd pups also suffer from ectopia lentis. Ectopia lentis is a rare condition in which the eye's lens is displaced from its normal position due to dysfunction of the zonular fibres (Sadiq and Vanderveen 2013; Simon et al. 2015). This condition can be traumatic, congenital (the form that probably existed in our patients), metabolic and consecutive or spontaneous (Gazi et al. 2024; Simon et al. 2015). Although no epidemiological study is available in veterinary medicine, it has been proposed that congenital ectopia lentis may have a prevalence of 6 per 100,000 in humans (Chandra and Charteris 2014). Complications of ectopia lentis can include displacement of the lens into the anterior chamber or the vitreous (which was present in our patients) or cataract formation (Sadiq and Vanderveen 2013), which resulted in complex microphthalmia in the Central Asian Shepherd pups. In our patients, the mean lens thickness was higher than that recorded in the healthy puppy, and the value reported in healthy beagle dogs of the same age (5.03 vs. 4.91 mm) (Maynard et al. 2014). This finding may be due to swelling of the cataractous lens, which probably led to an increase in the depth of the vitreous chamber of our patients (8.72 ± 1.15 mm) compared to the mean value recorded in the healthy puppy of our study (7.98 mm) and that reported in healthy mixed‐breed dogs of the same age (6.95 mm) (Paunksnis et al. 2001). It is worth noting that congenital cataracts can also be associated with microphthalmos, so a recessive inheritance mode for congenital cataracts and microphthalmia has been proposed in Miniature Schnauzer (Mellersh 2014; Saraiva and Delgado 2020). The cataractous lens may tear the zonules and lead to lens displacement. Following the displacement of the lens into the vitreous chamber, leakage of lens proteins can occur, causing vitritis and chorioretinitis (Sadiq and Vanderveen 2013). To investigate the etiology of retinal changes, histopathological examinations could be helpful, but due to the opposition of the dogs’ owner, enucleation of the eyes was not performed. Considering the possible complications caused by cataracts, the prognosis of these patients has been favourable so far. After 6 months, in a telephone follow‐up, the owner acknowledged that the puppies showed good physical and social development compared to their siblings.

The Central Asian Shepherd dog is one of the few dog breeds that do not have enough eye screening examination statistics. Therefore, the Genetics Committee of the American College of Veterinary Ophthalmologists has not been able to provide a detailed description of the hereditary eye diseases of this breed (ACVO Genetics Committee 2023). Ophthalmic disorders suspected to have a genetic basis are considered ‘known and presumed‐hereditary eye diseases’ until the genetic basis is well‐identified. An ophthalmic disorder is described as ‘presumed to be inherited’ when (A) it is diagnosed more frequently in a breed than in other breeds; (B) its occurrence increases in one breed; (C) it is diagnosed more regularly in dogs associated with a specific breed; (D) it has a characteristic feature; (E) it has a distinct age of onset and course of development; and (F) it is resembling an entity that has been affirmed to be inherited in another breed (Guandalini et al. 2018). However, assuming the possibility of heritability of microphthalmia in the Central Asian Shepherd dog, it should be noted that the small size of the population and the limited gene pool can contribute to the increased prevalence of a disease in a breed. This issue highlights the importance of further studies on congenital ocular disorders in this breed.

## 4 Conclusion

In the present report, bilateral complex microphthalmia was diagnosed in the Central Asian Shepherd puppies based on the ultrasonographically low value of the mean globe axial length and concurrent congenital ectopia lentis. The exact mechanism of the pathophysiology of these malformations is not well known. They can be related to a high grade of inbreeding in kennels, so breeding efforts should be aimed to confine such anomalies. In addition, the possible role of environmental and genetic factors cannot be ignored. Thus, it is suggested that comprehensive clinical screening plans be established so that dog breeders can screen for ‘presumed to be inherited’ disorders in a breed before breeding.

## Author Contributions

**Reza Azargoun**: project administration, resources, writing – review and editing. **Seyed Mohammad Hashemi‐Asl**: investigation, methodology, writing – review and editing. **Shokoufa Deldar**: visualization, writing – original draft, formal analysis.

## Conflicts of Interest

The authors declare no conflicts of interest.

## Peer Review

The peer review history for this article is available at https://www.webofscience.com/api/gateway/wos/peer‐review/10.1002/vms3.70494.

## Data Availability

The data that support the findings of this study are available from the corresponding author upon reasonable request.
